# Preparation of Aloe-Emodin Microcapsules and Its Effect on Antibacterial and Optical Properties of Water-Based Coating

**DOI:** 10.3390/polym15071728

**Published:** 2023-03-30

**Authors:** Nan Huang, Xiaoxing Yan

**Affiliations:** 1 Co-Innovation Center of Efficient Processing and Utilization of Forest Resources, Nanjing Forestry University, Nanjing 210037, China; 2 College of Furnishings and Industrial Design, Nanjing Forestry University, Nanjing 210037, China

**Keywords:** microcapsules, aloe-emodin, water-based coating, antibacterial property, optical property

## Abstract

With the development of science and technology, the function of waterborne coatings has been advanced to a higher standard, which requires researchers to innovate and expand the research on them. Aloe-emodin is a natural material with antibacterial properties. Applying its antibacterial effect to the coating can enrich its function and meet the diversified needs of consumers. In this study, the urea-formaldehyde resin was used as the wall material and the aloe-emodin as the core material to prepare the microcapsules. The coating rate, yield, and morphology of the microcapsules were characterized. Through an orthogonal experiment and a single factor experiment, the optimization scheme of microcapsule preparation was explored. The results indicated that the optimum preparation process of aloe-emodin microcapsules was as follows: the mass ratio of core material to wall material was 1:15, the molar ratio of urea to formaldehyde was 1:1.2, the temperature of microencapsulation was 50 °C, and the stirring speed of microencapsulation was 600 rpm. On this basis, the aloe-emodin microcapsules with 0%, 1.0%, 3.0%, 6.0%, 9.0%, and 12.0% contents were added to the waterborne coating to prepare the paint films, and their influence on the antibacterial and optical properties of the waterborne paint films was explored. The results demonstrated that the aloe-emodin microcapsules had antibacterial activity. When the content was 6.0%, the comprehensive performance of the film was better. The antibacterial rate of the film against *Escherichia coli* was 68.1%, and against *Staphylococcus aureus* it was 60.7%. The color difference of the film was 59.93, and the glossiness at 60° was 7.8%. In this study, the microcapsules that can improve the antibacterial performance of water-based coatings were prepared, which can expand the application of water-based coatings and provide a reference for the study of the functionalization of water-based coatings.

## 1. Introduction

With the improvement in material living conditions and the increased attention to health, the living environment is also receiving more and more attention [1,2]. Microbial pollution in the living environment will affect human health. Coatings are often used on furniture, utensils, and other household items and decoration [3,4,5,6,7,8], which people come into direct contact with in their daily life and work [9,10]. Bacteria may spread through the carrier of the coating on the object, so the coating with antibacterial properties is an effective way to block indirect transmission [11]. Antibacterial coatings can effectively reduce the bacterial density on furniture and other items, optimize the living environment, and have practical application value [12,13,14,15]. This requires us to explore the antibacterial function of the coating.

By wrapping the wall material around the surface of the core material, microcapsule technology creates tiny, spherical particles with a wall–core structure [16,17,18,19,20,21,22,23]. Using microcapsule technology to coat the core material with an antibacterial material can stabilize the properties of the core material, thus achieving a lasting antibacterial effect. If the antibacterial agent is formulated into microcapsules and then added to the coating, its performance is not only optimized, but it also provides the coating with an antibacterial function. This has practical significance for optimizing the preparation of the antibacterial agent and enriching the functionality of the coating.

Aloe-emodin is an anthraquinone compound derived mainly from *Aloe vera*, *Rheum palmatum* L., and other plants, and is an effective antibacterial ingredient in aloe [24]. It is extremely sensitive to *Staphylococcus* and *Streptococcus* and is a natural antibacterial agent. It not only effectively eliminates bacteria but is also safe and harmless without stimulation [25,26,27,28]. Aloe-emodin can clean up the hazardous metabolites secreted in the course of bacterial infection and the toxins that remain after the bacteria are killed, and it has a strong inhibitory effect on the synthesis of the nucleic acid and protein of *Staphylococcus* [29,30]. For instance, the α-toxin is an important cytotoxic factor released by *Staphylococcus aureus* and a key toxin for some infections. Aloe-emodin can effectively inhibit its activity and exert an antibacterial effect [31]. In addition to its good antibacterial properties, aloe-emodin is also often used as a raw material for cosmetic and hair care products. Thus, it will not cause harm to human skin when applied to coatings that are in direct contact with the human body. Moreover, the preparation of microcapsules using green and environmentally friendly materials is a hot spot of current research. However, the aloe-emodin itself is dark in color, its material state is orange-red crystal, and its particles are large and irregular in shape, so it is not suitable for direct addition to the coating. The microencapsulation technology can be applied to actual production by changing its processing performance, and it has broad development prospects.

In this study, the urea-formaldehyde resin was used as the wall material and the aloe-emodin as the core material, and the best technological parameters for preparing microcapsules were explored through the L_9_ (3^4^) orthogonal experiment and single factor experiment. The aloe-emodin microcapsules were mixed into the water-based coating with different loadings to prepare the paint films, and the microscopic morphology and chemical composition of the films were analyzed and investigated. The influence of the microcapsule loading on the antibacterial property against *Escherichia coli* and *Staphylococcus aureus*, optical properties, and roughness of the paint films was analyzed. The results provide a reference for the study of the antibacterial property of water-based coatings.

## 2. Materials and Methods

### 2.1. Test Materials

The materials involved in this test are listed in Table 1. The coating preparation mold was made of silica gel, and the size was 50 mm × 50 mm × 10 mm. The paint was Dulux water-based coating. The size of the polyethylene film was 40 mm × 40 mm × 0.08 mm. The diameter of the Petri dish was 90 mm. The *Escherichia coli* was the second-generation standard strain ATCC25922, and the *Staphylococcus aureus* was the second-generation standard strain ATCC6538 from the Beijing Biological Preservation Center.

### 2.2. Preparation Method of the Aloe-Emodin Microcapsules

In this study, an orthogonal experiment and a single factor experiment were used to explore the best process parameters for the synthesis of the microcapsules. First, the L_9_ (3^4^) orthogonal test was designed, as shown in Table 2, Table 3 and Table 4. By controlling the urea and formaldehyde molar ratio of n (urea):n (formaldehyde), the core–wall ratio of m (core material):m (wall material), the reaction temperature, and the stirring rate of the microencapsulation, a variety of microcapsules were obtained, and the process parameters with the greatest influence factors were analyzed and determined.

(1) Preparation of the wall material: after a specific amount of formaldehyde solution with a concentration of 37.0% and a specific amount of urea were mixed evenly, the triethanolamine was used to regulate the pH value to 8.0. Then the polyvinyl alcohol was added at a dosage of 1.0% of the mass of the urea. The solution was put into a magnetic stirrer at 80 °C and the stirring speed was set to 600 rpm. The wall material prepolymer was prepared after reaction for 1 h.

(2) Preparation of the core material: the sodium dodecylbenzene sulfonate was used as an emulsifier and mixed with water, and then stirred evenly. After that, the core material aloe-emodin was added and put into a magnetic stirrer. After the corresponding reaction temperature and stirring rate were set, the reaction lasted for 45 min, so that the aloe-emodin was fully emulsified.

(3) Microencapsulation: the prepared wall material was gradually poured into the prepared core material solution. The citric acid monohydrate crystal was used to regulate the pH value to 2.5, and the NaCl and SiO_2_ powder were added to give the wall material toughness and prevent the microcapsule particles from binding to each other. After continuous reaction for 2 h, the resulting solution was stored for 24 h. The product was filtered by ethanol and water several times, dried at 40 °C for 24 h, and then ground. The final orange powder was the aloe-emodin microcapsules.

On the basis of the orthogonal experiment, the single factor test was conducted on the factor that had the greatest influence in the orthogonal experiment to further optimize the preparation parameters of the microcapsule. The experimental materials are shown in Table 5.

### 2.3. Preparation Method of the Paint Films

The best microcapsules obtained from the single factor experiment were mixed with the water-based coating with contents of 0%, 1.0%, 3.0%, 6.0%, 9.0%, and 12.0%. The film without microcapsules was set as the control sample. The dosages of the films are shown in Table 6. The microcapsules and coatings were fully mixed and evenly coated in the mold, then dried and cured in an oven at 40 °C for 30 min until the quality was no longer changed, and then taken out. The paint film samples were gently removed from the mold to test the antibacterial and optical properties. In addition, these coatings with microcapsules were applied to the glass plate to test the roughness of the films.

### 2.4. Testing and Characterization

#### 2.4.1. Yield and Coverage Rate Test of the Microcapsules

The microcapsules obtained from the test were dried, weighed, and recorded, and the yield of microcapsules was obtained. The microcapsule powder with a weight of *m*_1_ was weighed and fully ground with a mortar until the wall material was damaged and the core material was exposed. The powder was put into the glassware, and the hot ethanol was added, so that the powder was soaked in it and placed in the 65 °C constant temperature environment for 2 h. The operation was repeated more than twice to fully dissolve the exposed core material with the ethanol. After soaking, the deionized water and ethanol were used to filter the powder, and the resulting material was dried in an oven at 40 °C, which comprised the residual wall material. The weight of the wall material was *m*_2_, and the coating rate of the microcapsule was calculated by Formula (1).

(1) $$P=\left(m_{1}-m_{2}\right)/m_{1}\times 100\%$$

#### 2.4.2. Micromorphology and Chemical Composition Test of the Microcapsules

The microcapsules were observed with an optical microscope and scanning electron microscope. The prepared aloe-emodin microcapsules were evenly spread on a slide and placed on the stage with the cover glass side facing up. After the spring clip was fixed, the focal length and brightness were adjusted, and the 20× lens was selected to observe and record the micromorphology of the microcapsules. The powder tablet press compressed the microcapsule powder into thin slices, and the infrared spectrometer characterized the chemical composition of the microcapsules and the paint films.

#### 2.4.3. Antibacterial Test of the Paint Films

*Escherichia coli* and *Staphylococcus aureus* were used in the antibacterial test. Based on GB/T 21866-2008 [32], the antibacterial property of the paint films was tested and recorded. First of all, the nutrient agar medium powder was added into purified water, heated, dissolved, and then sub-packed into Petri dishes to make several planar nutrient agar media for standby. The nutritional broth powder was added into purified water to make broth culture liquid. The NaCl was added into purified water and dissolved by heating to produce the elution solution with a concentration of 0.85%. The plane nutrient agar medium, broth medium, and elution solution were sterilized at 121 °C for 30 min. The polyethylene film was immersed in 70.0% ethanol solution for 30 min, flushed with the elution solution, and then dried for standby.

The slant preservation fungus was fresh fungus that had been preserved for no more than one month. The bacteria on the slant medium were transferred to the plane nutrient agar medium through the inoculation ring and cultured for 20 h in a constant temperature and humidity chamber at 37 °C. The 1–2 rings of fresh bacteria were taken from the plane nutrient agar medium and added to the broth culture medium. On the basis of GB/T 4789.2-2016 [33], the 1:1000 bacterial suspension was prepared with tenfold increasing diluents in sequence. A total of 0.5 mL of the bacterial suspension was dripped onto the prepared paint film, and the sterilized polyethylene film was picked up by a pair of tweezers and then covered on the paint film. The paint film to be tested was placed in a Petri dish and incubated in a constant temperature and humidity chamber with a temperature of 37 °C and a humidity of 98.0% for 24 h. Two groups of parallel tests were conducted for each sample. The samples cultured for 24 h were taken out, 20 mL of the elution solution was added, and the sample paint films and covering film were washed repeatedly. After the elution solution was stirred evenly, 0.5 mL of it was taken and inoculated into the plane nutrient agar medium and cultured in the same environment for 48 h.

The plane nutrient agar medium cultured for 48 h was taken out and put into the bacterial colony counter. Through careful observation, all the colonies displayed in the Petri dish were counted one by one with a pen. After counting, the data on the counter were the number of colonies in the Petri dish. The final result was the average of the two groups of parallel tests. The measured number of colonies was multiplied by 1000, which was the actual number of the viable bacteria recovered after 48 h of incubation. The antibacterial rate of the paint films was calculated by Formula (2). In it, *R* refers to the antibacterial rate, *B* refers to the average number of recovered colonies after 48 h for the film without microcapsules, and *C* refers to the average number of recovered colonies after 48 h for the films with microcapsules.

(2) $$R=\left(B-C\right)/B\times 100\%$$

#### 2.4.4. Optical Properties Test of the Paint Films

The color difference in the paint films was measured in line with GB/T 11186.3-1989 by a colorimeter [34]. The values of *L*, *a*, and *b* were noted during the test. *L* represents the brightness value of the paint films. A higher value indicates a brighter color of the paint film. *a* represents the red-green value of the paint films; a positive number indicates the color is reddish, and a negative number indicates the color is greenish. *b* refers to the yellow-blue value of the paint films. A positive number indicates the color is yellow, and a negative number indicates the color is blue. The test values of the film without microcapsules are *L*_1_, *a*_1_, *b*_1_, and the test values of the films with microcapsules are *L*_2_, *a*_2_, *b*_2_. The color difference Δ*E* was obtained by Formula (3). In it, Δ*a* = *a*_2_ − *a*_1_, Δ*L* = *L*_2_ − *L*_1_, Δ*b* = *b*_2_ − *b*_1_.

(3) $$\Delta E={\left[{\left(\Delta L\right)}^{2}+{\left(\Delta a\right)}^{2}+{\left(\Delta b\right)}^{2}\right]}^{1/2}$$

The glossmeter was used to measure the gloss of the paint films at 20°, 60°, and 85° based on GB/T 4893.6-2013 [35]. The gloss loss rate of the paint films at 60° was calculated based on Formula (4). *G_L_* is the loss rate, *G*_0_ is the gloss of the film without the microcapsules, and *G*_1_ is the gloss of the films mixed with microcapsules.

(4) $$G_{L}=\left(G_{0}-G_{1}\right)/G_{0}\times 100\%$$

The ultraviolet spectrophotometer was used to test the transmittance of the paint film, and the test wavelength range was 380–780 nm of visible light. After a beam of light passed through the sample, the ratio of the remaining light intensity to the incident light intensity was the transmittance.

#### 2.4.5. Tensile and Roughness Test of the Paint Films

The tensile test of the paint film was conducted with the universal mechanical testing machine. The stress–strain curve was drawn on the base of the test results. When the paint film broke, the ratio of the distance beyond the original length to the original length was the elongation at the break of the paint film.

The test method for the roughness of the paint film surface included placing the coated glass plate on the test table of the roughness meter and adjusting the position of the contact pin to contact the paint film to start the test and record the value of the roughness.

## 3. Results and Discussion

### 3.1. Analysis of the Yield and Coverage Rate of the Microcapsules

The yield of the microcapsules was a significant indicator in the performance evaluation of the microcapsules. To save resources and improve efficiency it was beneficial to prepare a high production of the aloe-emodin microcapsules with fewer crude materials.

**Figure 1:**
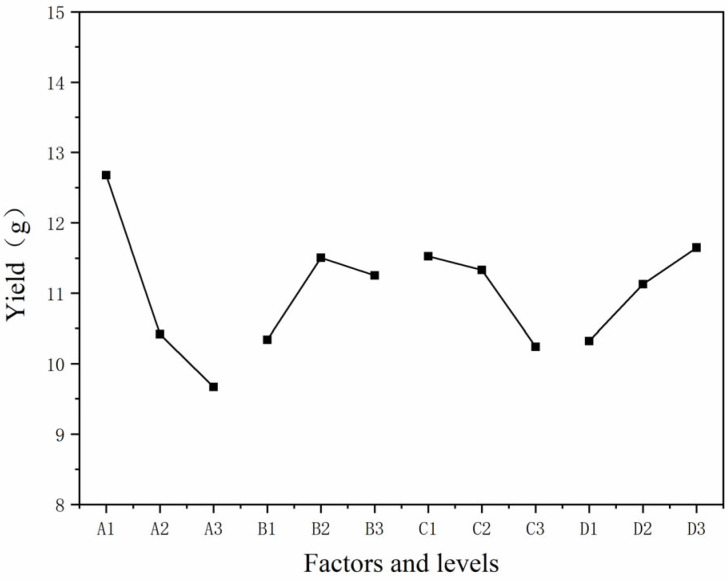
The visual analysis chart.

Table 7 reveals the analysis of the orthogonal test results of the microcapsule yield, and Figure 1 shows the visual analysis of each factor and level. The yield of sample 2 was the highest, reaching 13.55 g, followed by sample 1, reaching 11.77 g. Through a comparison of the mean value, the optimal level was determined to be A1B2C1D3. According to the comparison of the range results, the primary and secondary level of the influence of each factor on the microcapsule yield was A > D > C > B. The most influential factor was the molar ratio of urea and formaldehyde, followed by the stirring speed of microencapsulation, followed by the reaction temperature of microencapsulation, and finally the mass ratio of core material to wall material.

Table 8 is the variance analysis table of microcapsule yield results, and the variance results were basically consistent with the range results. The symbol “*” indicates that a factor is significant. It can be seen from the sum of squares data that factor A had the greatest impact on the yield results and was far more significant than the other three factors. The other factors had a small impact and a small gap. Based on the results of mean value, range, and variance, it can be seen that factor A, namely, the molar ratio of urea and formaldehyde, had the greatest impact on the yield. The better process parameter for preparing the urea-formaldehyde resin-coated aloe-emodin microcapsules was A1B2C1D3; that is, the molar ratio of urea and formaldehyde was 1:1.2, the mass ratio of core material to wall material was 1:20, the reaction temperature of microencapsulation was 50 °C, and the stirring speed of microencapsulation was 1200 rpm.

**Figure 2:**
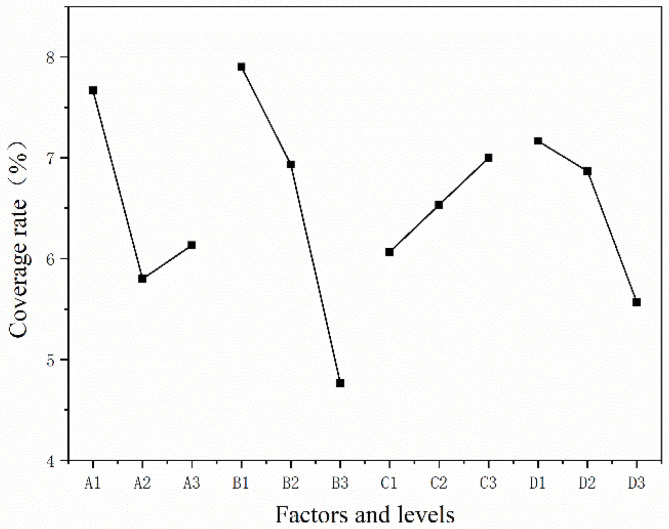
The visual analysis chart.

The coverage rate of the microcapsules was the ratio of the core material to the total microcapsule mass. The coverage rate of the core material in the microcapsules was the key to its antibacterial effect and was an important base for measuring the performance of the microcapsules. The mass of core material in this test was small, so the higher the coverage rate, the more successful the preparation of the aloe-emodin microcapsules. The mean value, range, and variance results of the coating rate of the microcapsules are shown in Table 9, Figure 2, and Table 10. The coating rate of sample 1 was the highest, up to 9.2%. By comparing the mean values, we inferred that the optimal level was A1B1C3D1. According to the comparison of the range data, the primary and secondary level of the influence of each factor on the coverage rate was B > A > D > C. The most influential factor was factor B, followed by factor A. The result of variance was roughly the same as the result of range. Comparing the data of the sum of squares, it was seen that factor B had the greatest impact on the microcapsule coverage rate, followed by factor A and factor D, and finally factor C. Combining the results of mean, range, and variance, it was deduced that factor B, namely, the mass ratio of core material to wall material, had the greatest impact on the coating rate, followed by factor A, namely, the molar ratio of urea and formaldehyde. The better process parameter for preparing the urea-formaldehyde resin-coated aloe-emodin microcapsules was A1B1C3D1; that is, the molar ratio of urea and formaldehyde was 1:1.2, the mass ratio of core material to wall material was 1:15, the reaction temperature of microencapsulation was 90 °C, and the stirring speed of microencapsulation was 600 rpm.

Through a comprehensive analysis of the orthogonal experiment results, the primary and secondary levels of the factors obtained from the yield test were found to be A > D > C > B, and the primary and secondary levels of the factors obtained from the coverage rate test were B > A > D > C. It can be inferred that the comprehensive impact of factor A was the largest. Considering that in the variance results of the yield test, factor A had the highest impact and factor B had the lowest impact, while factor A was only second to factor B in the coverage rate test, it was determined that among the four factors, factor A had the greatest impact on the yield and coverage rate of the microcapsules, namely, the molar ratio of urea and formaldehyde. The better process parameter obtained from the yield test was A1B2C1D3, and the better process parameter obtained from the coverage rate test was A1B1C3D1. The reaction temperature of C3 level was 90 °C, and the rotational speed of D3 level was 1200 rpm. Considering that, when the reaction temperature is too high and the rotational speed is too fast, the water in the solution will evaporate too quickly, affecting the accuracy of the test, the parameters of C1 and D1 levels were more suitable, at 50 °C and 600 rpm, respectively. Of the nine samples, sample 1 had the best morphology, highest coverage rate, and the second highest yield. Combined with the preparation parameter of this sample of A1B1C1D1, the better preparation process was determined to be A1B1C1D1: the molar ratio of urea and formaldehyde was 1:1.20, the mass ratio of core material to wall material was 1:15, the temperature was 50 °C, and the stirring speed was 600 rpm.

On the basis of these process parameters, in order to further study the effect of the molar ratio of urea and formaldehyde on the performance of the aloe-emodin microcapsules, the molar ratio of urea and formaldehyde was selected as a single factor variable to conduct a single factor test. In the test of yield and coverage rate, it was found that the parameter with the molar ratio of urea and formaldehyde set at 1:1.2 was the best. Considering that excessive formaldehyde will volatilize into the air and cause pollution, which does not conform to standards of health and environmental protection, in the single factor test, the molar ratio of urea and formaldehyde was controlled as follows: 1:1.00, 1:1.10, 1:1.15, 1:1.20, 1:1.25, 1:1.30, 1:1.35, 1:1.40, 1:1.50. Table 11 shows the corresponding yields and coverage rates of the microcapsules in the single factor test. These results indicated that the coverage rate of sample 12, sample 13, and sample 15 was the highest, exceeding 9.0%. Among them, sample 13, namely, the microcapsule with the molar ratio of urea and formaldehyde of 1:1.20, had the highest yield, up to 11.91 g, and was the best microcapsule sample in the single factor test.

### 3.2. Analysis of the Morphology of the Microcapsules

**Figure 3:**
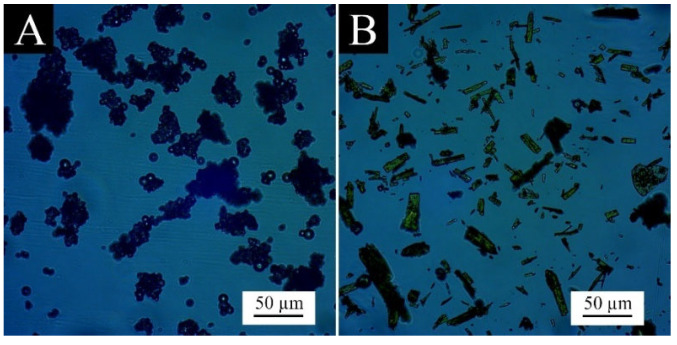
OM images of wall and core material: (**A**) wall material and (**B**) core material.

The micromorphology of the core material aloe-emodin powder and the urea-formaldehyde resin wall material powder prepared in this experiment is shown in Figure 3. Aloe-emodin appears as a long crystal because aloe-emodin itself is a large particle of dark orange needle-like crystal. The wall material appears as large irregular agglomerates because the urea-formaldehyde resin prepolymer itself has viscosity. After being ground into powder, the material particles are large, and there is still a large area of adhesion and aggregation.

**Figure 4:**
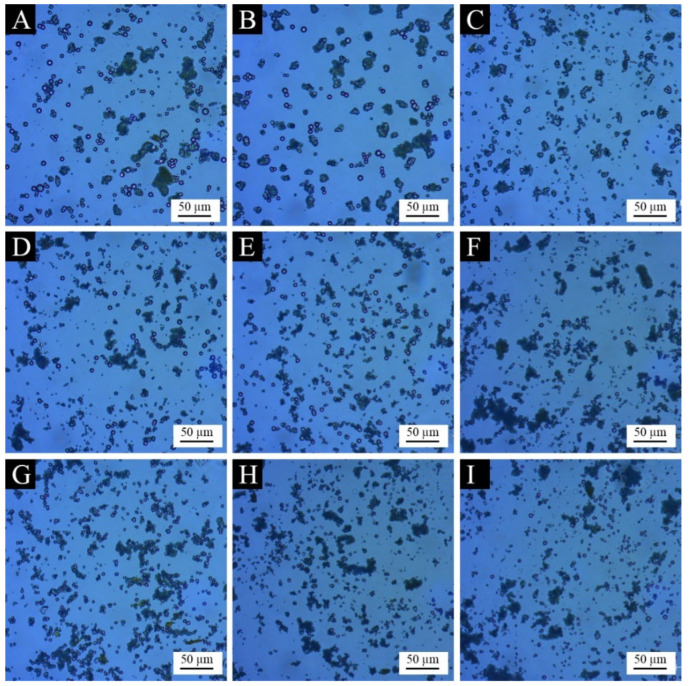
OM images of microcapsules in the orthogonal test: (**A**) sample 1, (**B**) sample 2, (**C**) sample 3, (**D**) sample 4, (**E**) sample 5, (**F**) sample 6, (**G**) sample 7, (**H**) sample 8 and (**I**) sample 9.

The micromorphology of microcapsules prepared from the orthogonal test is shown in Figure 4. Compared with the wall material and the core material, the prepared microcapsules had a significantly smaller particle size, presenting a spherical shape, basically without large agglomerates and needle-like crystals. It can be seen that the core material was well emulsified and dispersed, and the wall material was also more evenly coated around the core material. Among them, sample 1 and sample 2 had the best morphology, with more microcapsules and uniform dispersion and less agglomeration. The morphology of sample 6 and sample 9 was poor, and the microcapsules were fewer and had obvious aggregation. This may be because the mass ratio of the core material to the wall material of the two samples was 1:25, and the excessive wall material caused the agglomeration and deposition of the redundant urea-formaldehyde resin, which affected the formation and dispersion of the microcapsules.

**Figure 5:**
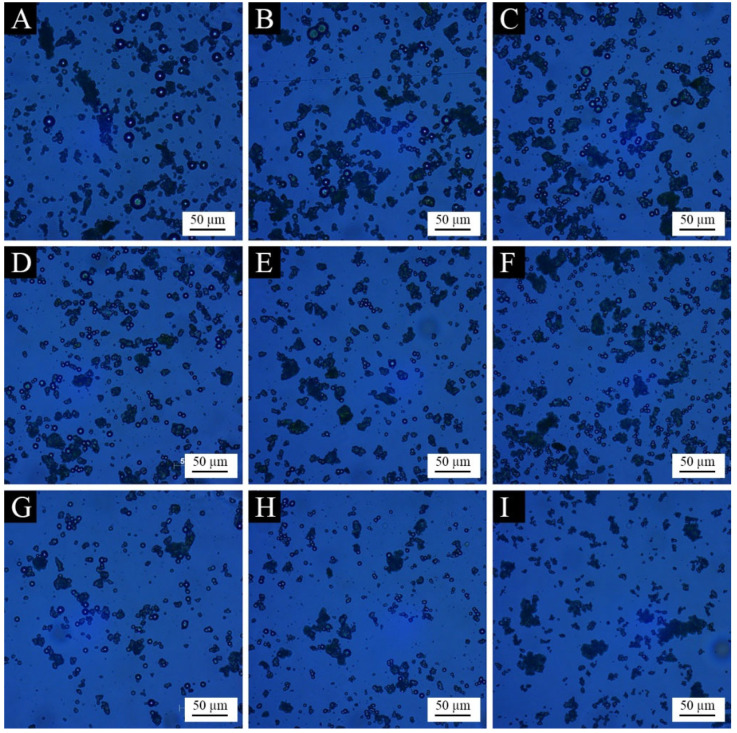
OM images of microcapsules in the single factor experiment: (**A**) sample 10, (**B**) sample 11, (**C**) sample 12, (**D**) sample 13, (**E**) sample 14, (**F**) sample 15, (**G**) sample 16, (**H**) sample 17, and (**I**) sample 18.

**Figure 6:**
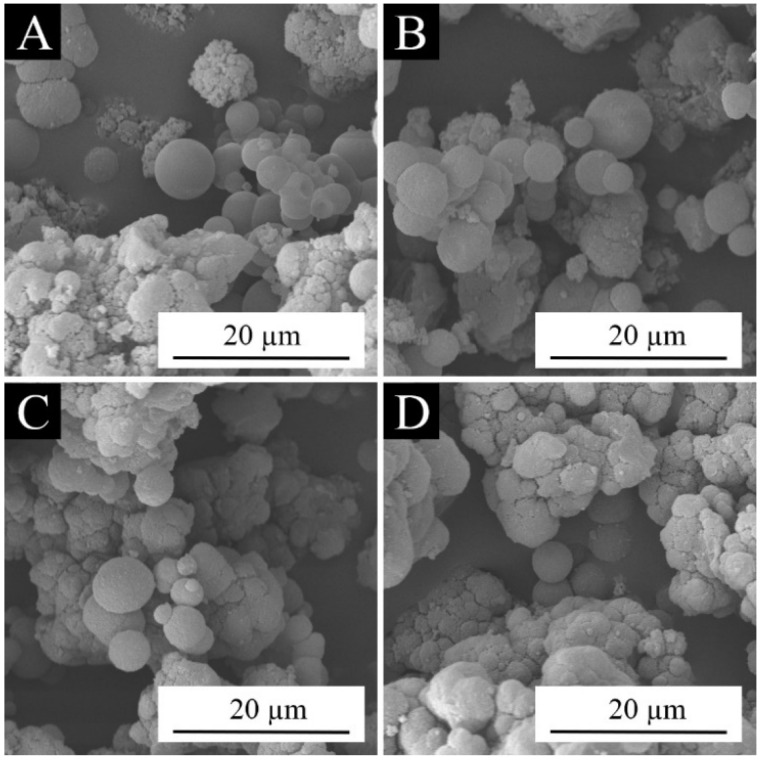
SEM images of microcapsules in the single factor experiment: (**A**) sample 10, (**B**) sample 13, (**C**) sample 15, and (**D**) sample 17.

Figure 5 shows the micromorphology of the microcapsules prepared in the single factor experiment. The morphology of samples 10–16 was good, and the microcapsules were mellow and evenly dispersed. Among them, the morphology of samples 12 and 13 prepared with the molar ratios of urea and formaldehyde of 1:1.15 and 1:1.20 was the best, which indicated that this parameter was better. The micromorphology of samples 17 and 18 was poor, as the molar ratios of urea and formaldehyde were 1:1.40 and 1:1.50, which indicated that excessive formaldehyde was not conducive to the formation of the microcapsules. Figure 6 is the SEM images of samples 10, 13, 15, and 17. In Figure 6A,B, we see that when the molar ratio of urea and formaldehyde was low, the microcapsules were spherical, with a smooth surface and a small amount of agglomeration. It can also be seen in Figure 6C,D that when the molar ratio was too high, although the microcapsules displayed a spherical shape, the phenomenon of adhesion was obvious, and there were many agglomerations.

Taking into account the microcapsule yield, coating rate, and micromorphology, sample 13 had the highest coating rate and yield, with uniform dispersion, smooth and round particles, and excellent morphology. Therefore, sample 13 was added to the water-based coatings with the mass fractions of 0%, 1.0%, 3.0%, 6.0%, 9.0%, and 12.0%, and the paint films were prepared by mold to determine the effect of the aloe-emodin microcapsules on the antibacterial and optical properties of the water-based coating.

### 3.3. Analysis of the Morphology of the Paint Films

**Figure 7:**
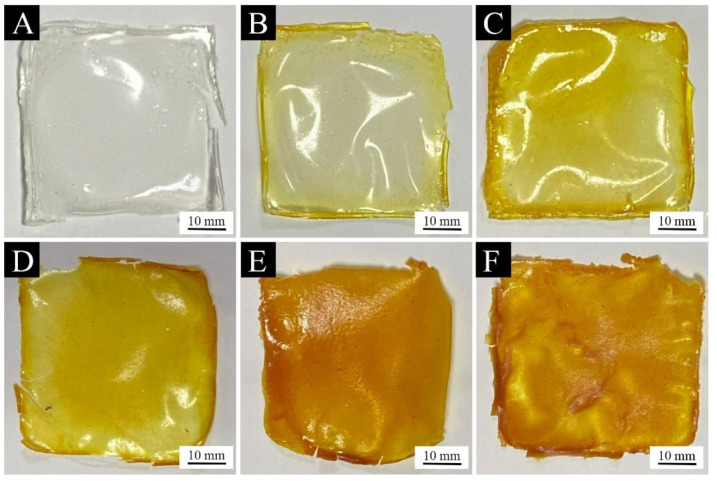
Macro morphology of the paint films: (**A**) 0% microcapsule-loaded film, (**B**) 1.0% microcapsule-loaded film, (**C**) 3.0% microcapsule loaded-film, (**D**) 6.0% microcapsule-loaded film, (**E**) 9.0% microcapsule-loaded film, and (**F**) 12.0% microcapsule-loaded film.

**Figure 8:**
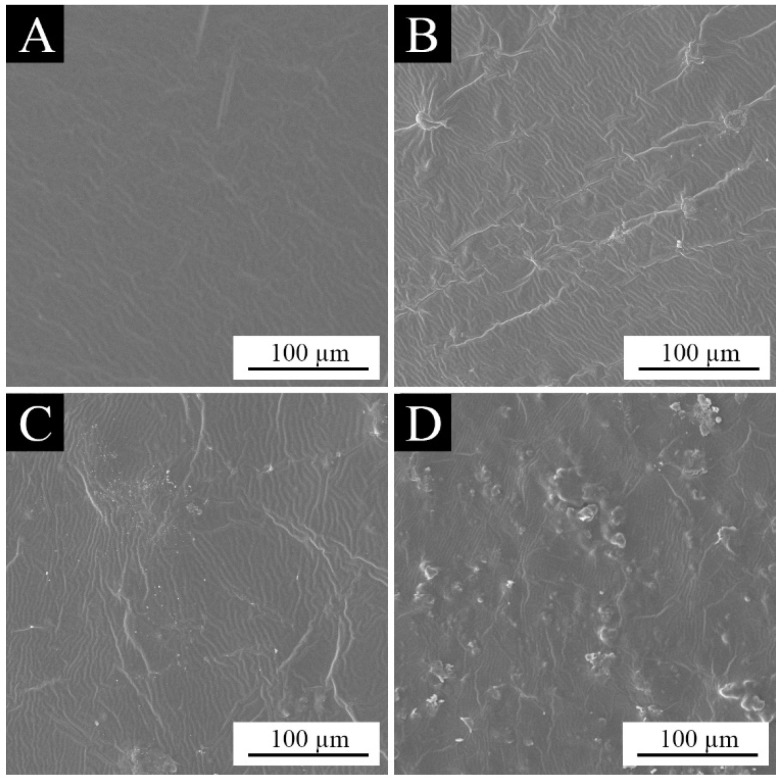
SEM images of the paint films: (**A**) 0% microcapsule-loaded film, (**B**) 3.0% microcapsule-loaded film, (**C**) 6.0% microcapsule-loaded film and (**D**) 12.0% microcapsule-loaded film.

Figure 7A–F shows the macro morphology of the paint films with 0%, 1.0%, 3.0%, 6.0%, 9.0%, and 12.0% aloe-emodin microcapsules added, respectively. It can be clearly observed from the figure that the film without microcapsule loading is colorless and transparent, and the films loaded with microcapsules are yellow and transparent. When the content was higher than 6.0%, it showed orange. With the increase in the aloe-emodin microcapsules, the color of the paint films gradually darkened and the transparency gradually decreased. This occurred because the microcapsules were orange and not transparent. With the increase in the loading, the average distribution of microcapsules in the coating increased, affecting the color and transparency of the paint films. In addition, Figure 7E shows that when the loading reached 9.0%, tiny cracks began to appear at the edge of the paint film. Figure 7F shows that when the loading was 12.0%, the cracks on the edge of the paint film led to an incomplete paint film, and the distribution of microcapsules in the coating was uneven, resulting in uneven color and uneven paint film. This occurred because the content of the aloe-emodin microcapsules was too high, resulting in a smaller content of the water-based coating and poor viscosity and leveling, indicating that the coating with 12.0% loading was not suitable for practical application. Figure 8A–D shows the microscopic morphology of the paint films with 0%, 3.0%, 6.0%, and 9.0% aloe-emodin microcapsules added, respectively. The paint film surface without microcapsule loading was level and smooth. When the loading was 3.0% and 6.0%, the paint film surface showed slight wrinkles. When the loading reached 9.0%, as shown in Figure 8D, in addition to wrinkles, there were many visible bumps on the paint film surface. This occurred because there were too many microcapsules, which could not be evenly dispersed in the coating, so they gathered together and produced large particles on the surface. It can be seen that the microcapsule content higher than 9.0% had a strong negative effect on the transparency and flatness of the paint films.

### 3.4. Infrared Spectrum Analysis of the Microcapsule and Paint Films

**Figure 9:**
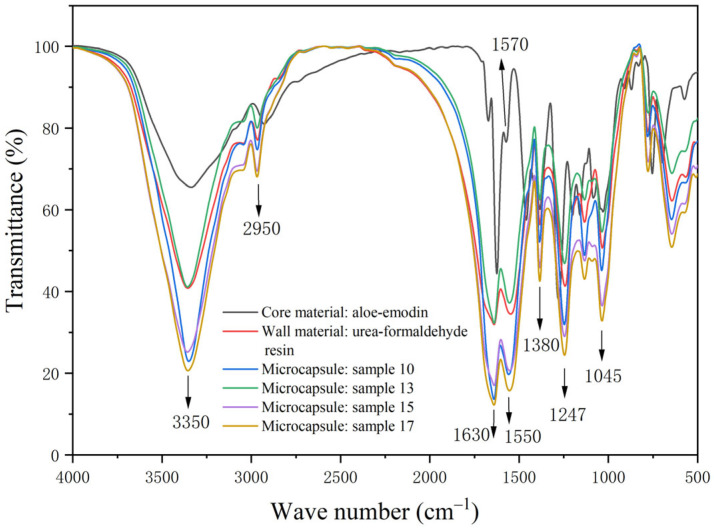
FTIR image of the core material, the wall material, and the microcapsules.

Figure 9 illustrates the absorption spectrogram of the aloe-emodin, the urea-formaldehyde resin, and the microcapsules. The peaks at 3350 cm^−1^, 2950 cm^−1^, and 1630 cm^−1^ are the stretching vibration peaks of O-H, the bending vibration peak of C-H, and the stretching vibration peak of C=O. These characteristic peaks were jointly possessed by the aloe-emodin and urea-formaldehyde resin and can also be clearly seen in the absorption curve of microcapsules. The peak at 1550 cm^−1^ is the characteristic peak of C-N in the urea-formaldehyde resin, and the peak at 1247 cm^−1^ was caused by the stretching vibration of C-N and the deformation vibration of N-H in the urea-formaldehyde resin. These were the characteristic peaks of the wall material and the microcapsules, indicating the existence of urea-formaldehyde resin in the microcapsules [36,37]. The peak at 1045 cm^−1^ is the C-O-C absorption peak of dimethylol urea in the urea-formaldehyde resin. The specific C=C in aloe-emodin appears at 1570 cm^−1^ [38], and this absorption peak also appears in the absorption curve of microcapsules, which indicated that there was a chemical component of aloe-emodin in the microcapsules. The above characteristic peaks proved that the microcapsules contained core material and wall material, and the chemical composition was not damaged, proving that the microcapsule coating was successful.

**Figure 10:**
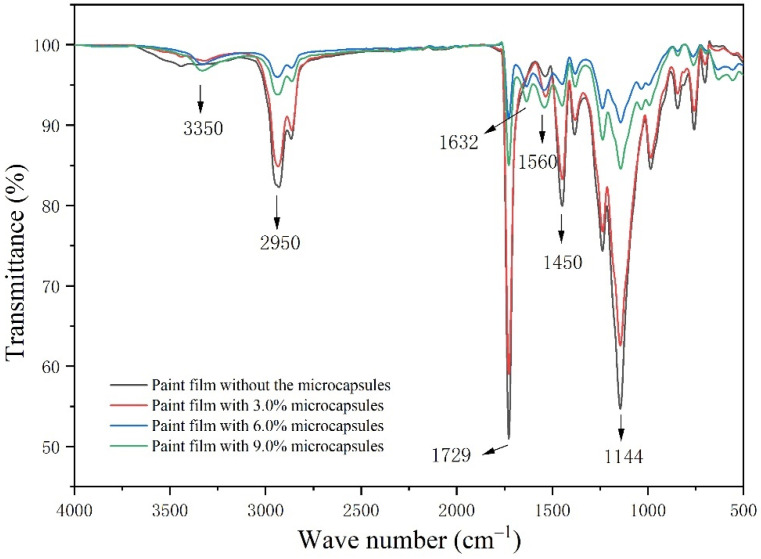
FTIR image of the paint films.

Figure 10 is the absorption spectrogram of the film without microcapsules and the films with microcapsules. The peaks at 3350 cm^−1^, 2950 cm^−1^, and 1144 cm^−1^ are the peaks of O-H, C-H, and C-O that were found in the water-based coating and the microcapsules. The characteristic peak at 1729 cm^−1^ belongs to the telescopic vibration peak of C=O in the water-based coating. The expansion vibration peak of (-CH_2_)-CH_2_ in the waterborne coating appears at 1450 cm^−1^. These characteristic peaks can be seen in the four curves, indicating that the chemical composition of the water-based coating was not damaged after mixing with the microcapsules. The 1560 cm^−1^ peak is characteristic of C=N in the microcapsule wall material, and the peak at 1632 cm^−1^ is the absorption peak of C=O in the microcapsule core material. These peaks can be seen in the curves of the films loaded with microcapsules, which proved that the chemical composition of the microcapsules in the water-based coating did not change. The existence of these characteristic peaks indicates that after the microcapsules were mixed with the water-based coating, there was no chemical reaction with the coating, and the components of the water-based coating and the microcapsules remained intact and effective.

### 3.5. Analysis of the Influence of Different Microcapsule Loading on the Antibacterial Property of the Paint Films

**Figure 11:**
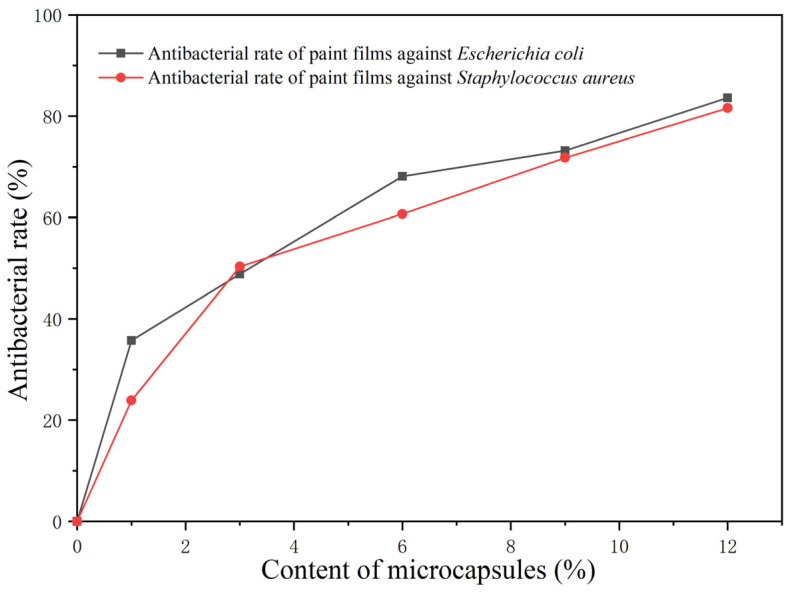
Antibacterial rates of the paint films.

**Figure 12:**
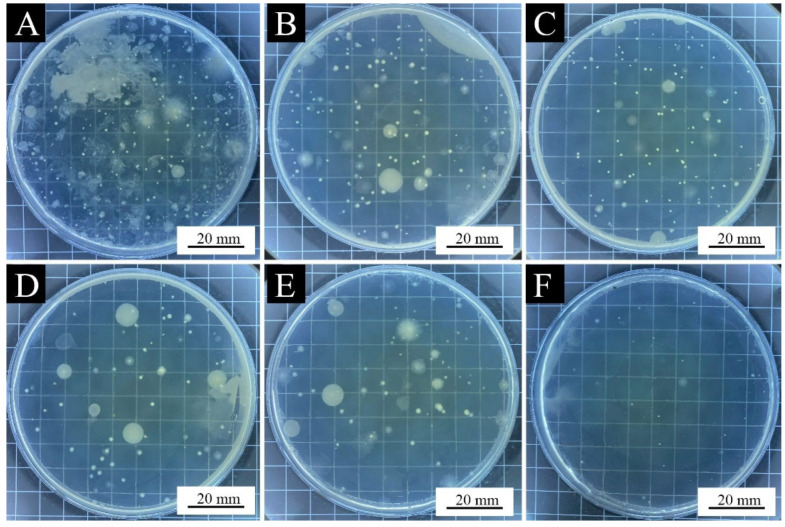
*Escherichia coli* colonies recovered after antibacterial test of the paint films: (**A**) 0% microcapsule-loaded film, (**B**) 1.0% microcapsule-loaded film, (**C**) 3.0% microcapsule-loaded film, (**D**) 6.0% microcapsule-loaded film, (**E**) 9.0% microcapsule-loaded film, and (**F**) 12.0% microcapsule-loaded film.

**Figure 13:**
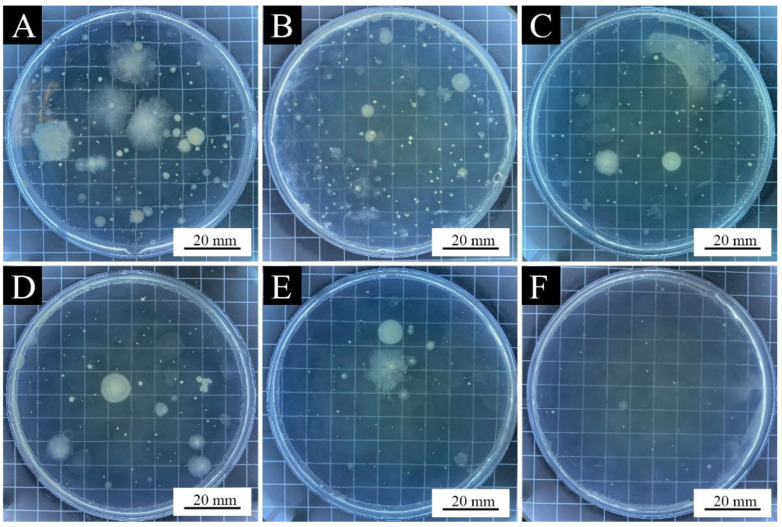
*Staphylococcus aureus* colonies recovered after antibacterial test of the paint films: (**A**) 0% microcapsule-loaded film, (**B**) 1.0% microcapsule-loaded film, (**C**) 3.0% microcapsule-loaded film, (**D**) 6.0% microcapsule-loaded film, (**E**) 9.0% microcapsule-loaded film, and (**F**) 12.0% microcapsule-loaded film.

Table 12 and Figure 11 show the actual number of colonies and the antibacterial rates of the paint films with different loadings of the aloe-emodin microcapsules after the antibacterial tests on the *Escherichia coli* and the *Staphylococcus aureus*. Figure 12 and Figure 13 are the bacterial colonies recovered after the antibacterial test. It can be seen from the data that with the increase in the loading, the antibacterial rate against both kinds of bacteria gradually increased, and the antibacterial effect gradually increased, reaching 83.6% and 81.6%, respectively. The antibacterial function of the paint films against the *Escherichia coli* was slightly better than that of the *Staphylococcus aureus*. When the loading was 6.0%, the antibacterial rate against the *Staphylococcus aureus* was slightly higher than against the *Escherichia coli*. These results indicated that the microcapsules successfully played an antibacterial role in the paint films. They showed that the aloe-emodin was microencapsulated and coated with urea-formaldehyde resin, which not only improved the problems of too-dark color and too-large particles, but also preserved its original antibacterial properties. This may have been because after drying, owing to the volatilization of the solvent, the wall material and the film produced tiny voids through which the aloe-emodin slowly inhibited the growth of the *Escherichia coli* and the *Staphylococcus aureus*.

### 3.6. Analysis of the Influence of Different Microcapsule Loading on the Optical Properties of the Paint Films

**Figure 14:**
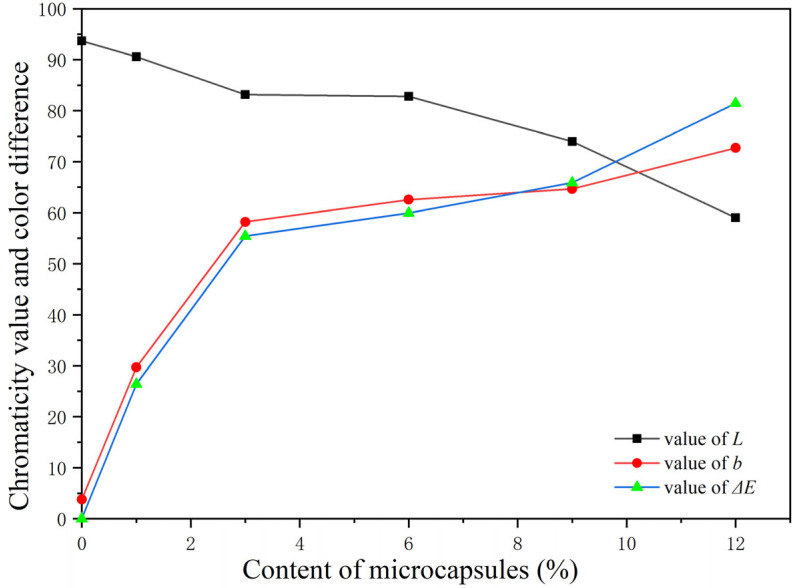
The trend of the chromaticity value and color difference value.

The chromaticity value and color difference of the paint films are indicated in Table 13 and Figure 14. With the increase in the aloe-emodin microcapsules, the color difference of the films loaded with the microcapsules compared with the film without microcapsules was on the rise. The *b* value indicating the yellow color also showed the same trend, which indicated that the yellow degree of the paint films became darker. This occurred because the microcapsule itself was yellow. On the contrary, the value of *L*, which represented the brightness, showed a downward trend. This occurred because the microcapsules were granular powder and did not chemically react with the coating. The state of existence of the coating was always granular. Therefore, after the addition of microcapsules to the coating, the density and ductility of the liquid coating itself were affected, resulting in a reduction in the surface smoothness and brightness of the dried paint films.

**Figure 15:**
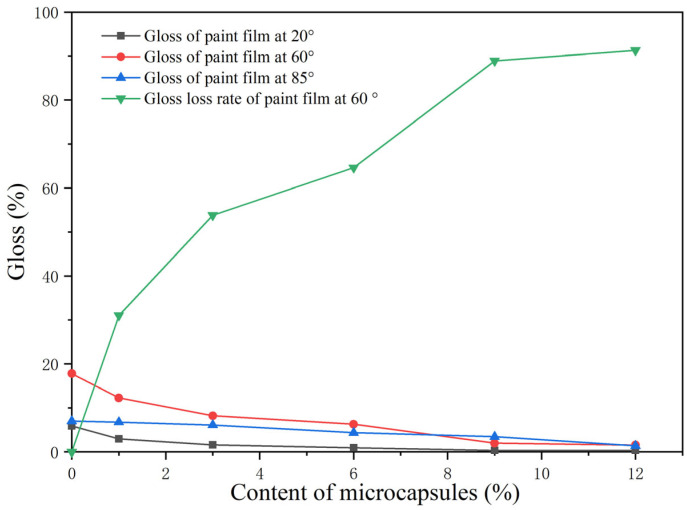
The trend of the gloss and gloss loss rate of the paint films.

The gloss and gloss loss rates of the paint films are indicated in Table 14 and Figure 15. As the microcapsule loading increased, the gloss gradually decreased, and the gloss loss rate at 60° gradually increased. When the loading reached 12.0%, the gloss was the lowest, and the loss rate was the highest, which was obviously not suitable for practical production applications. This occurred because the granular microcapsules affected the smoothness of the paint films after drying. When the content of microcapsules was high, the paint film surface was rough and the diffuse reflection of light was enhanced, so the gloss was very low.

**Figure 16:**
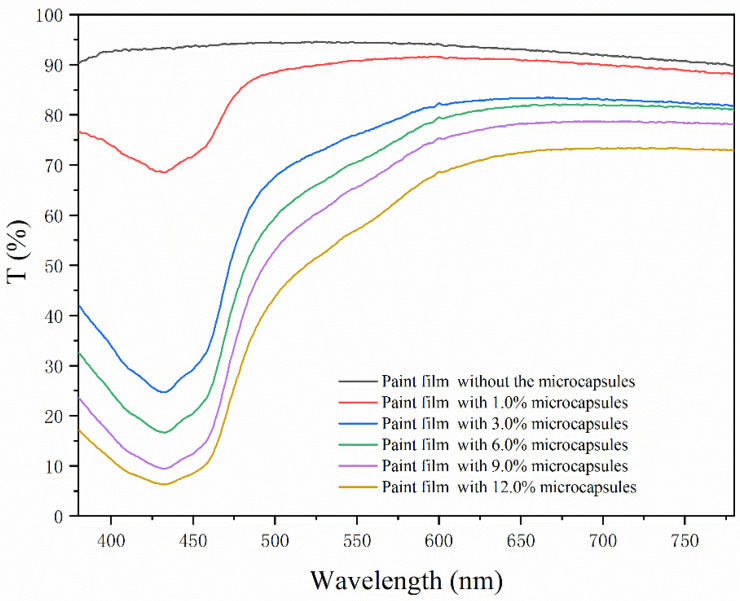
The trend of the transmittance of the paint films.

As shown in Figure 16, the transmittance of the paint film was negatively correlated with the loading of the aloe-emodin microcapsules. The higher the loading, the lower the transmittance. This occurred because the microcapsules were yellow and opaque, and adding them to the transparent water-based coating affected the light transmittance. As seen from the curve, in the wavelength range of 400–480 nm, the transmittance of the film with the microcapsules was far lower than that without the microcapsules, and the transmittance reached the lowest value at 430 nm. This occurred because the paint films with microcapsules were yellow, while purple and blue are the complementary colors of yellow. The wavelength range was respectively between 400–450 nm and 450–480 nm. When the visible light passed through the paint films, the purple light and blue light were absorbed, and the remaining light intensity was significantly lower than the incident light intensity, so the transmittance in this wave band was very low.

### 3.7. Analysis of the Influence of Different Microcapsule Loadings on the Tensile Resistance and Roughness of the Paint Films

**Figure 17:**
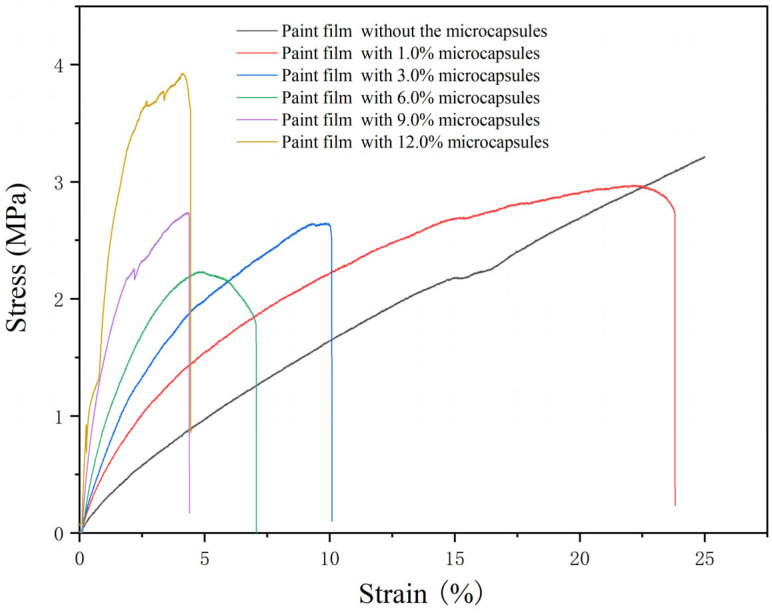
The stress–strain curves of the paint films.

The effect of the aloe-emodin microcapsules with different loadings on the tensile resistance of the paint films is shown in Figure 17. It can be seen from the figure that when the loading was less than 6.0%, the paint films had a certain elastic area, which decreased with the increase in the loading. This may be because the microcapsule particles were distributed in the paint film, which reduced the ductility of the water-based paint film itself. When the content reached 9.0% and 12.0%, the tensile area of the paint films decreased rapidly. At this time, the strain of the paint film under high stress was very small, which indicated that the paint film was strong and had low ductility. This phenomenon may have occurred because when the loading of the aloe-emodin microcapsules was too high, a large number of microcapsules were filled in the paint film, which enhanced its hardness and made it more brittle.

The elongation at the break of the paint film under different loadings is shown in Table 15. The elongation at the break decreased with the increase in the loading. When the loading was higher than 6.0%, the elongation at the break became very low. This occurred because the aloe-emodin microcapsules were dispersed in the paint film in the form of solid particles, which reduced the viscosity and elasticity of the paint itself. Therefore, when subjected to tensile force, the relative elongation of the paint film decreased and the elongation at the break decreased.

Table 16 indicates the roughness of the paint film under different microcapsule loadings. The roughness of the paint film was on the rise because the prepared microcapsules were spherical particles that were added to the water-based coating and then dried to prepare the paint film. The existing state in the paint film was granular. With the increasing addition, the particles increased, resulting in the uneven surface of the paint film and the increase in roughness.

## 4. Conclusions

The optimum preparation parameters of the aloe-emodin microcapsules were as follows: the molar ratio of urea and formaldehyde was 1:1.20, the mass ratio of core material to wall material was 1:15, the reaction temperature of microencapsulation was 50 °C, and the stirring speed of microencapsulation was 600 rpm. Based on the microcapsule yield, coverage rate, and micromorphology, it can be concluded that the microcapsule had the highest coverage rate and yield, up to 11.91 g and 9.0%, respectively, with uniform dispersion, smooth and round particles. The effect of the increase in microcapsule loading on the performance of the paint films was as follows: the antibacterial rates of the paint films against *Escherichia coli* and *Staphylococcus aureus* were gradually increased, with the maximum reaching 83.6% and 81.6%, respectively; the color difference was increased, with the maximum reaching 81.43; the glossiness was reduced; the light loss rate was increased; the light transmittance was reduced; the tensile property was reduced; the elongation at the break was decreased, with the minimum 4.1%; and the roughness was increased. When the loading of the microcapsules was as high as 12.0%, the paint film was no longer suitable for practical application. When the loading was 6.0%, the comprehensive performance of the paint film was more suitable. The antibacterial microcapsules and water-based coating were combined to successfully endow the coating with antibacterial function. This kind of coating has potential application value in the fields of furniture painting and architectural decoration.

## Figures and Tables

**Table 1:** List of the test materials.

Material	Molecular Formula	M_W_ (g/mol)	CAS No.	Concentration (%)
urea	CH_4_N_2_O	60.06	57-13-6	99.0
formaldehyde solution	-	-	-	37.0
triethanolamine	C_6_H_15_NO_3_	149.19	102-71-6	99.9
polyvinyl alcohol	[C_2_H_4_O]*_n_*	-	9002-89-5	99.0
sodium dodecyl benzene sulfonate	C_18_H_29_NaO_3_S	348.48	25155-30-0	99.9
aloe-emodin	C_15_H_10_O_5_	270.2369	481-72-1	98.0
citric acid monohydrate	C_6_H_10_O_8_	210.14	5949-29-1	99.9
anhydrous ethanol	C_2_H_6_O	46.07	64-17-5	99.9
sodium chloride	NaCl	58.4428	7647-14-5	99.5
silicon dioxide	SiO_2_	60.084	14808-60-7	99.5
waterborne acrylic resin	-	-	9003-01-4	-
*Escherichia coli*	-	-	-	-
*Staphylococcus aureus*	-	-	-	-
nutrient agar medium	-	-	-	-
nutritional broth	-	-	-	-
polyethylene film	-	-	-	-
Petri dish	-	-	-	-

**Table 2:** Formulation scheme of the orthogonal experiment.

Level	Factor A n (Urea):n (Formaldehyde)	Factor B m (Core Material):m (Wall Material)	Factor C Temperature (°C)	Factor D Stirring Speed (rpm)
1	1:1.2	1:15	50	600
2	1:1.5	1:20	70	900
3	1:1.8	1:25	90	1200

**Table 3:** Preparation parameters of the orthogonal experiment.

Sample	Factor A n (Urea):n (Formaldehyde)	Factor B m (Core Material):m (Wall Material)	Factor C Temperature (°C)	Factor D Stirring Speed (rpm)
1	1:1.2	1:15	50	600
2	1:1.2	1:20	70	900
3	1:1.2	1:25	90	1200
4	1:1.5	1:15	70	1200
5	1:1.5	1:20	90	600
6	1:1.5	1:25	50	900
7	1:1.8	1:15	90	900
8	1:1.8	1:20	50	1200
9	1:1.8	1:25	70	600

**Table 4:** Material list of the orthogonal experiment.

Sample	Urea (g)	Formaldehyde Solution (g)	Wall Material (g)	Polyvinyl Alcohol (g)	Aloe-Emodin (g)	Deionized Water (g)	Emulsifier (g)	NaCl (g)	SiO_2_ (g)
1	10.00	16.22	16.00	0.10	1.07	232.65	2.35	1.28	1.28
2	10.00	16.22	16.00	0.10	0.80	174.24	1.76	1.28	1.28
3	10.00	16.22	16.00	0.10	0.64	139.59	1.41	1.28	1.28
4	10.00	20.27	17.50	0.10	1.17	255.42	2.58	1.40	1.40
5	10.00	20.27	17.50	0.10	0.88	191.07	1.93	1.40	1.40
6	10.00	20.27	17.50	0.10	0.70	152.46	1.54	1.40	1.40
7	10.00	24.32	19.00	0.10	1.27	276.21	2.79	1.52	1.52
8	10.00	24.32	19.00	0.10	0.95	213.84	2.16	1.52	1.52
9	10.00	24.32	19.00	0.10	0.76	165.33	1.67	1.52	1.52

**Table 5:** Material list of the single factor test.

Sample	Urea (g)	Formaldehyde Solution (g)	Wall Material (g)	Polyvinyl Alcohol (g)	Aloe-Emodin (g)	Deionized Water (g)	Emulsifier (g)	NaCl (g)	SiO_2_ (g)
10	10.00	13.51	15.00	0.10	1.00	217.80	2.20	1.20	1.20
11	10.00	14.86	15.50	0.10	1.03	224.73	2.27	1.24	1.24
12	10.00	15.54	15.75	0.10	1.05	227.70	2.30	1.26	1.26
13	10.00	16.22	16.00	0.10	1.07	232.65	2.35	1.28	1.28
14	10.00	16.89	16.25	0.10	1.08	234.63	2.37	1.30	1.30
15	10.00	17.57	16.50	0.10	1.10	239.58	2.42	1.32	1.32
16	10.00	18.24	16.75	0.10	1.12	243.54	2.46	1.34	1.34
17	10.00	18.92	17.00	0.10	1.13	245.52	2.48	1.36	1.36
18	10.00	20.27	17.50	0.10	1.17	254.43	2.57	1.40	1.40

**Table 6:** Dosage list of the water-based paint films.

Content of the Microcapsules (%)	Microcapsule Weight (g)	Coating Weight (g)
0	0	1.400
1.0	0.014	1.386
3.0	0.042	1.358
6.0	0.084	1.316
9.0	0.126	1.274
12.0	0.168	1.232

**Table 7:** Analysis of microcapsule yield results.

Sample	Factor A n (Urea):n (Formaldehyde)	Factor B m (Core Material):m (Wall Material)	Factor C Temperature (°C)	Factor D Stirring Speed (rpm)	Yield (g)
1	1:1.2	1:15	50	600	11.77
2	1:1.2	1:20	70	900	13.55
3	1:1.2	1:25	90	1200	12.72
4	1:1.5	1:15	70	1200	10.97
5	1:1.5	1:20	90	600	9.72
6	1:1.5	1:25	50	900	11.56
7	1:1.8	1:15	90	900	8.28
8	1:1.8	1:20	50	1200	11.25
9	1:1.8	1:25	70	600	9.48
Mean value 1	12.680	10.340	11.527	10.323	
Mean value 2	10.417	11.507	11.333	11.130	
Mean value 3	9.670	11.253	10.240	11.647	
Range	3.010	1.167	1.287	1.324	
Primary and secondary order	A > D > C > B				
Optimal level	A1	B2	C1	D3	
Optimal scheme	A1 B2 C1 D3				

**Table 8:** Analysis table of variance.

Sources of Variation	Quadratic Sum	Free Degree	F-Ratio	F-Critical Value	Significance
Factor A	13.951	2	2.564	4.460	*
Factor B	2.259	2	0.415	4.460	
Factor C	2.888	2	0.531	4.460	
Factor D	2.669	2	0.490	4.460	
Error	21.77	8			

**Table 9:** Analysis of microcapsule coverage rate results.

Sample	Factor A n (Urea):n (Formaldehyde)	Factor B m (Core Material):m (Wall Material)	Factor C Temperature (°C)	Factor D Stirring Speed (rpm)	Coverage Rate (%)
1	1:1.2	1:15	50	600	9.2
2	1:1.2	1:20	70	900	8.4
3	1:1.2	1:25	90	1200	5.4
4	1:1.5	1:15	70	1200	6.2
5	1:1.5	1:20	90	600	7.3
6	1:1.5	1:25	50	900	3.9
7	1:1.8	1:15	90	900	8.3
8	1:1.8	1:20	50	1200	5.1
9	1:1.8	1:25	70	600	5.0
Mean value 1	7.667	7.900	6.067	7.167	
Mean value 2	5.800	6.933	6.533	6.867	
Mean value 3	6.133	4.767	7.000	5.567	
Range	1.867	3.133	0.933	1.600	
Primary and secondary order	B > A > D > C				
Optimal level	A1	B1	C3	D1	
Optimal scheme	A1 B1 C3 D1				

**Table 10:** Analysis table of variance.

Sources of Variation	Quadratic Sum	Free Degree	F-Ratio	F-Critical Value	Significance
Factor A	5.947	2	0.880	4.460	
Factor B	15.447	2	2.285	4.460	*
Factor C	1.307	2	0.193	4.460	
Factor D	4.340	2	0.642	4.460	
Error	27.04	8			

**Table 11:** Microcapsule yields and coverage rates in the single factor experiment.

Sample	n (Urea):n (Formaldehyde)	Yield (g)	Coverage Rate (%)
10	1:1.00	11.59	8.4
11	1:1.10	11.78	7.3
12	1:1.15	11.27	9.1
13	1:1.20	11.91	9.3
14	1:1.25	11.51	6.8
15	1:1.30	11.11	9.1
16	1:1.35	11.32	8.1
17	1:1.40	11.21	7.9
18	1:1.50	9.76	6.4

**Table 12:** Antibacterial rates of the paint films with different loadings of the microcapsules.

Content of the Microcapsules (%)	Average Number of Recovered *Escherichia coli* (CFU·piece^−1^)	Antibacterial Rate Against *Escherichia coli* (%)	Average Number of Recovered *Staphylococcus aureus* (CFU·piece^−1^)	Antibacterial Rate Against *Staphylococcus aureus* (%)
0	213	-	163	-
1.0	137	35.7	124	23.9
3.0	109	48.8	81	50.3
6.0	68	68.1	64	60.7
9.0	57	73.2	46	71.8
12.0	35	83.6	30	81.6

**Table 13:** Chromaticity value and color difference value of the paint films.

Content of the Microcapsules (%)	*L*	*a*	*b*	Δ*E*
0	93.64 ± 0.7	−0.77 ± 0.7	3.78 ± 0.4	-
1.0	90.57 ± 1.5	−4.67 ± 2.5	29.71 ± 0.9	26.40
3.0	83.17 ± 0.7	−1.03 ± 1.6	58.20 ± 2.8	55.41
6.0	82.82 ± 2.6	3.86 ± 1.6	62.54 ± 1.2	59.93
9.0	73.95 ± 2.4	14.99 ± 2.5	64.65 ± 1.2	65.88
12.0	59.01 ± 2.6	25.39 ± 2.1	72.69 ± 1.3	81.43

**Table 14:** Gloss and extinction rates of the paint films.

Content of the Microcapsules (%)	20° (%)	60° (%)	85° (%)	Gloss Loss Rate (%)
0	93.64 ± 0.7	−0.77 ± 0.7	3.78 ± 0.4	-
1.0	90.57 ± 1.5	−4.67 ± 2.5	29.71 ± 0.9	26.40
3.0	83.17 ± 0.7	−1.03 ± 1.6	58.20 ± 2.8	55.41
6.0	82.82 ± 2.6	3.86 ± 1.6	62.54 ± 1.2	59.93
9.0	73.95 ± 2.4	14.99 ± 2.5	64.65 ± 1.2	65.88
12.0	59.01 ± 2.6	25.39 ± 2.1	72.69 ± 1.3	81.43

**Table 15:** Elongation at the break of the paint films.

Content of Microcapsules (%)	Elongation at Break (%)
0	31.2
1.0	22.2
3.0	9.9
6.0	4.9
9.0	4.3
12.0	4.1

**Table 16:** Roughness of the paint films.

Content of Microcapsules (%)	Roughness (μm)
0	0.13 ± 0.1
1.0	0.55 ± 0.2
3.0	0.89 ± 0.1
6.0	1.54 ± 0.1
9.0	2.14
12.0	2.67 ± 0.2

## Data Availability

The data presented in this study are available on request from the corresponding author.
